# Sex differences in HIV testing among elders in Sub-Saharan Africa: a systematic review protocol

**DOI:** 10.1186/s13643-022-01968-7

**Published:** 2022-05-16

**Authors:** Akalewold T. Gebremeskel, Olumuyiwa Omonaiye, Sanni Yaya

**Affiliations:** 1 Faculty of Health Sciences, University of Ottawa, Ottawa, Ontario Canada; 2 School of International Development and Global Studies, University of Ottawa, Ottawa, Ontario Canada; 3 Centre for Quality and Patient Safety Research, School of Nursing and Midwifery, Deakin University, Burwood, Melbourne, Victoria Australia; 4 Centre for Nursing and Midwifery Research, James Cook University, Townsville, Queensland Australia; 5 The George Institute for Global Health, Imperial College London, London, UK

## Abstract

**Background:**

Elders (age 50+) HIV demographic (age and sex) data are essential to better understand their HIV service utilization and develop appropriate evidence-based responses and policies. Despite a significant prevalence rate of HIV and growing numbers of this population group, data are still scarce, and studies have neglected them in Sub-Saharan Africa. The aim of this protocol is to outline the methodological process of a systematic review that will gather qualitative and quantitative data to critically examine sex differences in HIV testing among elders (age 50+) in Sub-Saharan Africa.

**Methods:**

This protocol adheres to the PRISMA-P reporting guidelines. We will conduct a systematic database search to retrieve all observational and qualitative studies. Electronic search strategies will be developed for MEDLINE, EMBASE, Web of Science, Global Health, and CINAHL for studies reporting HIV data. Two reviewers will independently screen all citations, full-text articles, and abstract data. The search strategy will consist of free-text and Medical Subject Headings (MeSH) terms. Search terms for elders (50+) will include the following: “elders”, “older adults”, “aged”, “geriatric” and “seniors”. The primary outcome of interest is sex differences in the uptake of HIV counselling and testing (HCT). The study methodological quality (or bias) will be appraised using appropriate tools. Screening, data extraction, and assessments of risk of bias will be performed independently by two reviewers. Narrative synthesis will be conducted with studies that are compatible based on population and outcome. As it will be a systematic review, without human participants’ involvement, there will be no requirement for ethical approval.

**Discussion:**

The systematic review will present key evidence on sex differences in HIV testing among elders in Sub-Saharan Africa. The findings will be used to inform program developers, policymakers, and other stakeholders to enhance sex disaggregated HIV data to improve access to HIV counselling and testing service for elders in Sub-Saharan Africa. The final manuscript will be disseminated through a peer-reviewed journal and scientific conferences.

**Systematic review registration:**

PROSPERO: CRD42020172737.

**Supplementary Information:**

The online version contains supplementary material available at 10.1186/s13643-022-01968-7.

## Background

The Sustainable Development Goals (SDGs) adopted in 2015 requires sex-disaggregated data to track progress towards leaving no one behind [1]. The SDGs specifically targeted to achieve healthy lives and well-being at all ages, and gender equality. In this study elders are defined as people aged 50 years and older. We used the cut-off of 50 years and older because most of the literature including publications from UNAIDS on HIV/AIDS in older adults defines older as ≥ 50 years of age [2–5]. In addition, several human immunodeficiency (HIV)-related research conducted in Sub-Saharan Africa have used the cut-off point of aged 50 years and over to define elders or older adults [6, 7].

In 2015, there were 46 million people 60 years or older in Sub-Saharan Africa, an increase from 23 million in 1990 [8]. According to the Department of Economic and Social Affairs, Population Division of the United Nations, it is projected that 161 million older persons will reside in the region by 2050 [8]. This growth rate is faster than that experienced by any other region since 1950 [8].

In the context of HIV, it is estimated that there are three million adults 50 years and older living with HIV in Sub-Saharan Africa representing over 14% of those over the age of 15 infected [9]. Despite this, most HIV messaging and prevention interventions in Sub-Saharan Africa is targeted toward persons between ages 15 and 49 years [9–11]. Thus, most research on HIV in the continent have largely ignored aged 50 and older [10–12]. HIV testing and counseling (HCT) is a vital component for HIV control-HCT is the entry-point to prevention, care, and treatment which is required to reduce mortality and halt ongoing HIV transmission [13]. There are different modalities of HCT, which include standalone voluntary counseling center/clinic, mobile and home-based, and health facility integrated. The primary goal of home-based HCT is to bring HCT services to households, overcoming some of the impediments of access to HCT services and providing testing to individuals who might not otherwise seek services [14]. Facility integrated HCT entail integrating HCT services into existing different point of services within a health facility to ensure that all clients have access to HCT whenever they are accessing care and treatment within the health facility [15].

HCT has been shown to influence positive behaviour change for those found to be infected with HIV [16–18]. Hence, HCT is a key component of public health efforts to lower HIV incidence [19]. However, evidence shows that older adults in Sub-Saharan Africa lag behind in accessing HCT service by comparison to other age groups and are less likely to have been tested for HIV [9, 20, 21]. This is because limited HIV prevention intervention is targeted at older adults [9], healthcare providers may overlook testing older adults for HIV [22] and older adults may have low levels of awareness of the risk for contracting HIV [23]. Consequently, older adults are disadvantaged when it comes to accessing early diagnosis of HIV infection and timely initiation of antiretroviral therapy [24–26]. Thus, given the necessity for early identification of HIV infection among older patients, it is crucial that healthcare professionals adopt routine HIV screening as a standard of care for this population group regardless of their previous HIV negative status [27].

Furthermore, one of the major challenges of public health programmes including HIV control, is inequalities in health among different groups [28]. Gender/sex, race/ethnicity/culture/language, place of residence, religion, occupation, education, and socio-economic status are examples of some of the different determinants of health inequalities. Additionally, in this current era of large scale up of HCT initiatives in Sub-Saharan Africa [29], clarifying sex differences in HIV testing among older adults may give insights about their testing experiences and how their HIV care needs can be effectively met. However, there is paucity of data about the role that sex difference play regarding the uptake of HIV testing in Sub-Saharan Africa among older adults. This information is crucial as it will provide information about the prevalence and testing behaviour of older adults.

Additionally, elders (age 50+) HIV demographic (age and sex) data are essential to better understand their HIV service utilization and develop appropriate evidence-based responses and policies. To track gaps among women and men on various social and economic indicators, it is critical that data are disaggregated by sex and age. This is because sex and age-related data are crucial to understanding how HIV may impact different population groups and whether programs are meeting the unique needs of such groups [30]. Sex- and age-disaggregated data are also essential to the efficient and effective use of limited public health resources [31]. Therefore, we hypothesize that there will be sex differences in HCT uptake among elders in Sub-Saharan Africa because research demonstrates that in Sub-Saharan Africa the odds of ever having an HIV test is smaller for adults aged 50 years and over and smaller for older females compared to males aged 25–49 [9]. Additionally, in Sub-Saharan Africa lower testing rates have been recorded among older adults and among older women in particular [9]. Furthermore, we suspect that certain social norms and biological factors may be at play that could be responsible for sex differences in HCT uptake in this region. For example, late diagnosis and commencement of antiretroviral therapy is widespread among midlife-older men, who subscribe to long-established views of masculinity and age which view accessing HIV services, including testing, as a threat to their status in community and family life [32, 33]. For some midlife-older women, who no longer require family planning or antenatal care services, access to and uptake of HIV testing decreases substantially [34].

Thus, the review will include and analyze data disaggregated by sex and age. Analysis of data by sex and age also help to ensure that health systems do not perpetuate inequalities which are linked with undesirable health outcomes [30].

Therefore, the aim of this protocol is to outline the methodological process of a systematic review that will gather qualitative and quantitative data to examine sex differences among elders (age 50+) in Sub-Saharan Africa.

## Methods

### Protocol registration and reporting

This protocol has been registered within the PROSPERO database (the registration number is CRD42020172737). This protocol was designed and written according to the Preferred Reporting Items for Systematic Review and Meta-analysis Protocols (PRISMA-P) guideline for reporting systematic reviews, (see checklist in Additional file 1) [35]. This review will be conducted as per the Cochrane Handbook for Systematic Reviews of Interventions [36] and the findings will be reported in accordance with the reporting guidance provided in the PRISMA statement [37].

### Type of study

Observational and qualitative studies will be included in the review. Eligible studies must have reported sex differences in HIV testing uptake in a standard of HCT service among older adults in Sub-Saharan Africa.

### Type of participants

Studies focusing on elders (50+) men and women who received HCT in Sub-Saharan Africa will be considered.

### Type of intervention(s)

There is no specific intervention targeted for this study, however, we will consider standard HCT services including stand alone, mobile, home to home and health facility-based services.

### Type of outcome

The primary outcome of interest is sex differences in the uptake of HCT and factors associated with the differential uptake among elders (age 50+) in Sub-Saharan Africa.

### Inclusion criteria

Studies that report sex disaggregated data in HCT utilization either in health facility or community settings among elders (age 50+) in Sub-Saharan Africa will be included. Elders HCT utilization is defined as elders (50+) men and women who received an HIV test and counselling and who know their HIV test result in Sub-Saharan Africa. Articles published between January 2000 and January 2020 will be considered. This period is selected because the years 2000 to 2015 represent the era of Millennium Development Goals, where significant progress was made around the world, including in infants’ health. As a continuation of the increased focus of research on child health, the Sustainable Development Goals adopted in 2015 set new targets to reduce health inequality by 2030. The period from 2000 to 2020 thereby accounts for the new wave of research related to development goals on health for all, since the turn of the century.

### Exclusion criteria

HTC studies conducted outside of Sub-Saharan Africa countries, non-primary literature, such as reviews, editorials, study protocols and clinical guidelines, non-peer reviewed journal articles, and conference proceedings will be excluded.

### Search methods for identification of studies

#### Electronic searches

The primary source of the literature will be a structured search of major electronic databases (from inception onwards): MEDLINE, EMBASE, Web of Science, Global Health, and CINAHL for studies reporting data on HCT uptake among elders. The searches will be designed and conducted by the review team which includes two experienced public health researchers in collaboration with a Health Sciences librarian. We will perform hand-searching of the reference lists of included studies, relevant reviews, and other relevant documents such as government reports and grey literature. Examples of key words that will be used in the search include elders or senior or aged or geriatric, HIV or HIV/AIDs, counseling, and testing or screening. A draft search strategy for multiple databases is provided in Additional file 2 and a sample of a line-by-line strategy that has been tested in Medline is provided in Additional file 3.

### Data collection and analysis

#### Selection of studies

The articles retrieved from searches in each database will be uploaded into the Covidence article online management system to be screened by two authors (AG&OO) within the Covidence database for their relevance and eligibility to the review. The two reviewers will independently screen the titles and abstracts according to a pre-defined inclusion criteria checklist and will exclude unrelated ones. In case of disagreement between the reviewers, the judgment of article inclusion in the study will be made by a third person. The full texts will be read by the two individuals separately, and the final decisions will be made based on the checklist of inclusion criteria. The PRISMA statement (Preferred Reporting Items for Systematic Review and Meta-Analyses) flowchart will be used to document the selection process [37].

#### Data extraction and management

The authors will adapt a data collection form based on the needs of the review from a standardized data extraction form by the Cochrane library. The data extracted will include all details specific to the review question, fulfilling the requirements for the narrative synthesis. The content of each included studies will be extracted by two team members, independently and potential conflicts will be resolved through discussion. A data extraction form will be designed and used to extract information from each study report. Information of interest will include the following:

1. Study characteristics: study design, year of publication, journal, year (or period) of study conduct, and geographical location of where study was conducted, and factors associated with HCT will also be extracted.
2. Participant characteristics**:** sample size, age, and sex/gender.
3. Model of HCT: stand alone HCT clinic, mobile/home to home HCT, and health facility integrated HCT modalities.
4. Data collection method (e.g., survey, facility-based record)
5. Statistical analyses method and main outcomes including elders HCT utilization sex disaggregated data.

We will also contact primary study authors for key information when data are ambiguous or missing from the included studies.

#### Appraisal of study quality

Methodological rigor in this review will be achieved by having two independent reviewers critically appraising the methodological validity of the included studies. The reviewers will evaluate the qualitative studies using the Critical Appraisal Skills Programme checklists (CASP) qualitative checklist: 10 questions to help you make sense of a Qualitative research [38]. Overall narrative description of the findings of the appraisal of the qualitative studies will be presented. Quantitative studies will be evaluated with Quality Assessment Tool for Quantitative Studies [39] by the Effective Public Health Practice Project [39]. Each study will be assessed based on the six domains and given a score of ‘strong’, ‘moderate’ or ‘weak.’ Then an overall score will be given for each study based on the sum of values given on the six domains. Global rating for the studies will be presented as ‘strong’ (no weak ratings), ‘moderate’ (one weak rating) and ‘weak’ (two or more weak ratings). Studies will not be excluded or weighted based on the quality of the reporting assessments. The results of the appraisals and assessments will be used to inform data interpretation and ultimately determine trustworthiness of review findings and conclusions.

Two authors will independently check all the publications for completeness and accuracy of the quality assessment. Differences in decisions relating to the quality assessment will be resolved by discussion among all the authors.

#### Data synthesis

The data from each paper (e.g., study characteristics, context, participants’ age and sex, outcomes, and conclusion) will be used to build evidence tables of an overall description of included studies. Narrative methods of synthesis will be used to synthesize both quantitative and qualitative studies. It is defined as ‘an approach to the systematic review and synthesis of findings from multiple studies that relies primarily on the use of words and text to summaries and explain the findings of the synthesis [40] This approach is equally suitable for both quantitative and qualitative studies, as the emphasis is on an interpretive synthesis of the narrative findings of research. It will allow us to encompass the cross disciplinary and methodologically pluralistic natures of research in this topic area of HCT uptake gender difference and associated factors among aged (50+).

## Discussion

In Sub-Saharan Africa, there is an increasing widespread availability of HCT services because of the implementation of several intervention strategies such as home-based HIV testing [41], couples testing during antenatal care visits [42], provider-initiated testing, and counselling [43]. In general, despite the increased availability of HCT services, perceived psychological burden of having to live with HIV, stigma, and gender inequality are some of the factors that remains a significant barrier to the uptake of HCT services [44, 45]. Specifically, among younger population groups in Sub-Saharan Africa, obstacles to HCT include but not limited to lack of awareness of available services, low perception of personal risk, fear of negative consequences related to a positive test result, concerns about confidentiality, and lack of HIV/AIDS knowledge [45, 46]. Limited data is available on HCT among elders (age 50+) specifically [21]. Hence, the dearth of information on testing behaviour of elders (age 50+) may result in a high rate of undiagnosed, untreated, and late diagnosed HIV [21]. This might lead to poorer prognosis due to severely compromised immune system at start of antiretroviral treatment (ART) and increased risk of mortality [21]. Therefore, this review will provide insight on the extent to which health system sex disaggregated data collection may have impacted sex inequality in elders access to HCT. Thus, this systematic review will summarize the evidence regarding context, and participants’ sex and age differences in relation to the uptake and utilization of HCT services among elders in Sub-Saharan Africa. This knowledge may also provide insights about the burden of HIV among older adults which can potentially help to expand targeted prevention activities and testing services -thus leading to a robust public health-oriented HIV epidemic control. Additionally, in this current era of large scale up of HCT initiatives in Sub-Saharan Africa [29], clarifying sex differences in HIV testing among older adults may give insights about their testing experiences and how the their HIV care needs can be effectively met.

Program managers, policy-makers, and researchers can use the findings to enhance access to HCT services for elders in Sub-Saharan Africa. This systematic review and its evidence synthesis will be published in a peer-reviewed journal and presented at different conferences and scientific meetings.

We foresee limited studies reporting sex disaggregated data on HCT for elders. This is because the few existing studies have reported older people as single category, typically including all adults aged 50 years and over [47]. Furthermore, potential limitation may also include elders’ age and sex data which are unrecorded, underreported, and misreported. The aforementioned issues can result into missing information (reporting bias), methodological quality issues, and lack of studies that have assessed the elder’s sex disaggregated HCT uptake in Sub-Saharan Africa.

## Supplementary Information

**Additional file 1.** PRISMA-P 2015 Checklist.

**Additional file 2.** Electronic search strategies.

**Additional file 3.** Sample of Search Strategy used in Medline.

## Data Availability

Data sharing is not applicable to this article as no datasets were generated or analyzed during the current study.

## Abbreviations

HIV: Human immunodeficiency virus
PRISMA-P: Preferred Reporting Items for Systematic Review and Meta-analysis Protocols
PROSPERO: International Prospective Register of Systematic Reviews
SDGs: Sustainable Development Goals
CASP: Critical Appraisal Skills Programme checklists
