# Supplementary material for: Sex differences in HIV testing among elders in Sub-Saharan Africa: a systematic review protocol
Source: Syst Rev. 2022 May 16;11:95. doi: 10.1186/s13643-022-01968-7 (PMC9109370; doi:10.1186/s13643-022-01968-7)
Supplement: Supplementary file 2 — Additional file 2. Electronic search strategies. [file 13643_2022_1968_MOESM2_ESM.docx]

**Supplementary File 2**

**Search terms**

**Set 1: Setting**

Sub-Saharan Africa or Angola or Benin or Botswana or Burkina Faso or Burundi or Cameroon or Cape Verde or Central African Republic or Chad or Congo or Ivory Coast or Djibouti or Eritrea or Ethiopia or Gabon or Gambia or Ghana or Guinea or Kenya or Lesotho or Liberia or Madagascar or Malawi or Mali or Mauritania or Mauritius or Mozambique or Namibia or Niger or Nigeria or Reunion or Rwanda or Senegal or Seychelles or Sierra Leone or Somalia or South Africa or Sudan or Swaziland or Tanzania or Togo or Uganda or Zambia or Zimbabwe

**Set 2: Population**

1. *MeSH*

exp elders, HIV testing and counselling/ or exp elders, seniors / or exp elders, ageing/ or exp elders, geriatric/ or exp elders, mature adults/ or exp elders, aged/ or (HIV or HIV/AIDs) adj2 counseling and testing, screening

1. *Keywords*

Elders or senior or aged or geriatric

HIV or HIV/AIDs

counseling and testing or screening

**Search Summary (Ovid)**

Set 1 and Set 2 (A or B)
